# Immersion ultrasonography improves the repeatability of cephalic vein diameter measurements for inexperienced operators

**DOI:** 10.1080/0886022X.2022.2131573

**Published:** 2022-10-14

**Authors:** Zhijun Zhang, Shu He, Hui Wang, Yu Zhong, Hairong Zou, Xuan Gao

**Affiliations:** a Department of Ultrasound, University-Town Hospital of Chongqing Medical University, Chongqing, China; b Chongqing Renji Hospital, University of Chinese Academy of Sciences, Chongqing, China

**Keywords:** Immersion ultrasonography, cephalic vein, repeatability, nephrology training, ultrasound training, procedure training

## Abstract

**Objective:**

To reduce the empirical dependence of ultrasound measurement of the cephalic vein diameter, improve the repeatability of measurements for inexperienced operators, and provide a new method for inexperienced operators.

**Methods:**

Operators without ultrasound experience used contact ultrasound and immersion ultrasound to measure the diameter of the cephalic vein. The intraobserver and interobserver repeatability of measurements obtained *via* the two methods were analyzed.

**Results:**

The intraobserver and interobserver repeatability of the cephalic vein diameter measured *via* contact ultrasound by inexperienced operators were average, with intraclass correlation coefficients (ICCs) of 0.572 (95% CI: 0.239–0.759) and 0.405 (95% CI: −0.057–0.666), respectively. The intraobserver and interobserver reproducibility of the cephalic vein diameter measured by immersion ultrasound were very good, with ICCs of 0.955 (95% CI: 0.922–0.975) and 0.943 (95% CI: 0.900–0.967), respectively. In the Bland–Altman diagram of the intraobserver and interobserver agreement of the immersion ultrasound measurements of the cephalic vein diameter, 96% of the points fell within the 95% limits of agreement.

**Conclusion:**

Immersion ultrasonography can be used to measure the cephalic vein diameter while reducing the dependence of the results on operator experience; inexperienced operators can achieve very good repeatability.

It is very important to evaluate the cephalic vein diameter before surgery for radiocephalic arteriovenous fistulas (AVFs), as this diameter is related to the success rate of fistula creation and the patency rate in the later period [1]. Preoperative ultrasound is an effective method for measuring the cephalic vein [2]. Studies have shown that a cephalic vein diameter >2 mm is associated with a higher fistula creation success rate [3,4], while a cephalic vein diameter <1.6 mm may be associated with fistula creation failure [5]. Ultrasound is effective in the preoperative evaluation of AVFs. However, experienced operators are needed to ensure the accuracy and repeatability of the measurements [6]. In previous reports, measurements have been conducted by experienced operators, and the repeatability of these measurements has been found to be highly dependent on operator experience [4,7]. However, methods to improve the repeatability of operator measurements, especially those of inexperienced operators, and the factors affecting measurement repeatability have not been reported. The aims of this study were to reduce the empirical dependence of ultrasound measurement of the cephalic vein diameter and to improve the repeatability of measurements by inexperienced operators.

## Materials and methods

### Research objects

This study was conducted at the University-Town Hospital of Chongqing Medical University. Approval for this study was obtained from the hospital ethics committee (approval number LL-202118), and informed consent was obtained from the patients.

Early ultrasound examination revealed no stenosis, variations, inflammation or other diseases in the upper extremity vessels of the participants. The exclusion criteria were as follows: history of upper extremity vessel fistulas, history of trauma, and skin scarring.

### Instruments and equipment

A Canon APLIO400 color Doppler ultrasound instrument was used for ultrasound measurement. A 12 MHz linear array probe was selected. A transparent rectangular plastic box 80 cm long, 30 cm wide and 20 cm high was used for immersion ultrasonography. Room-temperature physiological saline was used as the coupling medium.

### Method of measurement

The two operating physicians had no actual ultrasound experience but could identify blood vessels on ultrasound images. Before the test, the two operators were trained on the use of the ultrasound equipment for 1 h so that they could complete the inspection independently.

Two-dimensional ultrasound was performed for cephalic vein measurement; measurements were made on transverse sections from one side of the vein wall to the other side of the wall.

**Figure 1.**
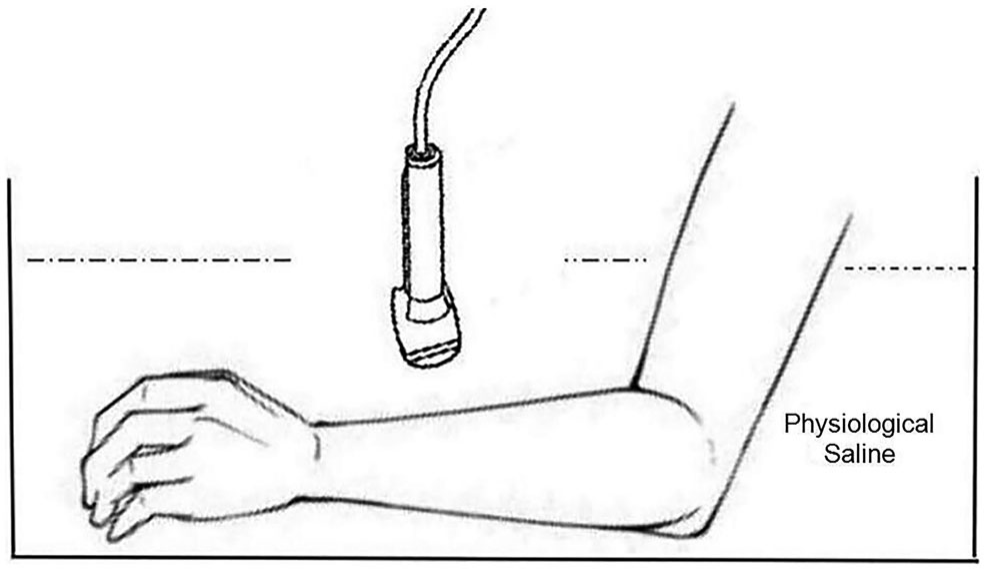
Schematic diagram of cephalic vein diameter measurement by immersion ultrasonography.

For each of the 48 patients, a line was drawn 2 cm above the radial styloid process of the left hand. Contact ultrasound was performed by applying ultrasound gel to the mark and lightly placing the probe on the skin for cephalic vein examination. The ultrasound probe has full contact with the skin to ensure a clear visualization of the cephalic vein (zero distance between the ultrasound probe and the skin). Immersion ultrasound was performed by adding room-temperature physiological saline to the tank to a fluid level 2 cm above the wrist. The patient’s elbow joint was kept at 90°, and the ultrasound probe was kept perpendicular to the skin during the scanning process. The ultrasound probe, with a protective cover, was then immersed in the saline; the probe was maintained at a distance of approximately 1 cm from the skin and did not make skin contact (Figure 1). During all inspections, the room temperature remained the same, and the subjects had a normal body temperature and normal heart rate.

At different time points, the two operators used contact ultrasound and immersion ultrasound to perform ultrasound examination of the cephalic vein in the 48 patients, measuring the diameter of the cephalic vein at the mark. The specific operation was as follows: operator 1 used contact ultrasound to measure the diameter of each cephalic vein. This was the first measurement result of operator 1. Then, operator 2 used the contact ultrasound method to obtain the first measurement result of operator 2. After resting for 10 min, operators 1 and 2 repeated the abovementioned contact ultrasound inspection to obtain the second contact ultrasound measurement results. Four sets of data were obtained. After 30 min of rest, the two operators used immersion ultrasound to measure the internal diameter of the cephalic vein in all subjects. The process was the same as that described above, and four sets of immersion ultrasound measurement data were obtained. The two operators were not involved in the data analysis.

### Statistical methods

IBM SPSS Statistics 25 was used to evaluate the intraobserver and interobserver repeatability of the two methods. The definition of repeatability was as follows: intraclass correlation coefficient (ICC) <0.4, poor; 0.40–0.60, average; 0.60–0.75, good; and 0.74–1.00, very good [8]. Bland–Altman analysis using MedCalc statistical software was also performed to evaluate the reproducibility. *p* < 0.05 was considered statistically significant.

## Results

The subjects were 48 patients requiring AVF creation and included 28 males and 20 females (age, 56–88 years [average, 72 ± 5.2 years]; weight, 45–65 kg [average, 52 ± 6.1 kg]; height, 155–175 cm [average, 165 ± 6.5 cm]; and BMI, 20.8–25.7 [average, 23.8 ± 3.6]). All 48 patients had end-stage renal disease. The primary diseases included chronic glomerulonephritis in 20 patients, diabetic nephropathy in 18 patients, and hypertensive nephropathy in 10 patients.

Table 1 shows that the intraobserver and interobserver reproducibility of contact ultrasound were average, with ICCs of 0.572 (95% CI: 0.239–0.759) and 0.405 (95% CI: −0.057–0.666), respectively.

**Table 1.** Repeatability of cephalic vein diameter measurements by contact ultrasonography (*n* = 48).

Parameter	Operator 1 (1st)	Operator 1 (2nd)	Operator 2 (1st)	Intraobserver ICC (95% CI)	Interobserver ICC (95% CI)
Diameter (mm)	2.12 ± 0.14	2.16 ± 0.18	2.14 ± 0.16	0.572 (0.239–0.759)	0.405 (−0.057–0.666)

ICC: intraclass correlation coefficient; CI: confidence interval.

Table 2 shows that the intraobserver and interobserver reproducibility of immersion ultrasound were very good, with ICCs of 0.955 (95% CI: 0.922–0.975) and 0.943 (95% CI: 0.900–0.967), respectively.

**Table 2.** Repeatability of cephalic vein diameter measurements by immersion ultrasonography (*n* = 48).

Parameter	Operator 1 (1st)	Operator 1 (2nd)	Operator 2 (1st)	Intraobserver ICC (95% CI)	Interobserver ICC (95% CI)
Diameter (mm)	2.28 ± 0.20	2.29 ± 0.18	2.27 ± 0.18	0.955 (0.922–0.975)	0.943 (0.900–0.967)

ICC: intraclass correlation coefficient; CI: confidence interval.

**Figure 2.**
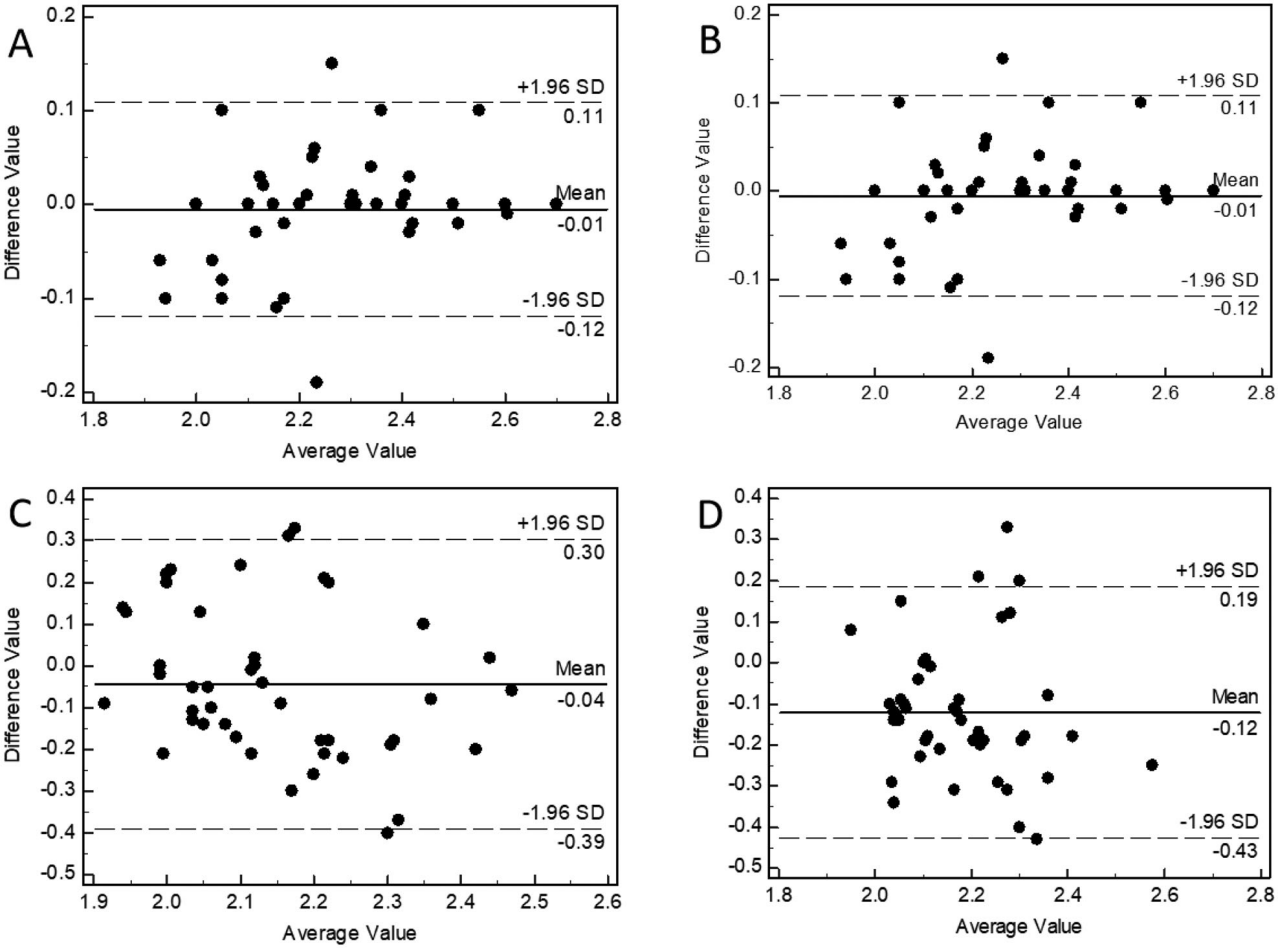
Bland–Altman plot of cephalic vein diameter measured by inexperienced operators using two separate methods. (A) Intraobserver Bland–Altman plot; immersion ultrasonography. (B) Interobserver Bland–Altman plot; immersion ultrasonography. (C) Intraobserver Bland–Altman plot; contact ultrasonography. (D) Interobserver Bland–Altman plot; contact ultrasonography.

In the Bland–Altman diagram of the intraobserver and interobserver results of immersion ultrasound in measuring the cephalic vein diameter, 96% of the points fell within the 95% limits of agreement (Table 3, Figure 2).

**Table 3.** Intraobserver and interobserver Bland–Altman plot analysis of cephalic vein diameter measured by immersion ultrasonography (*n* = 48).

Bland‒Altman analysis results	Intraobserver repeatability	Interobserver repeatability
Mean difference (mm)	−0.005	0.017
95% CI	−0.022–0.011	−0.001–0.036
*p*	0.536	0.069

CI: confidence interval.

**Figure 3.**
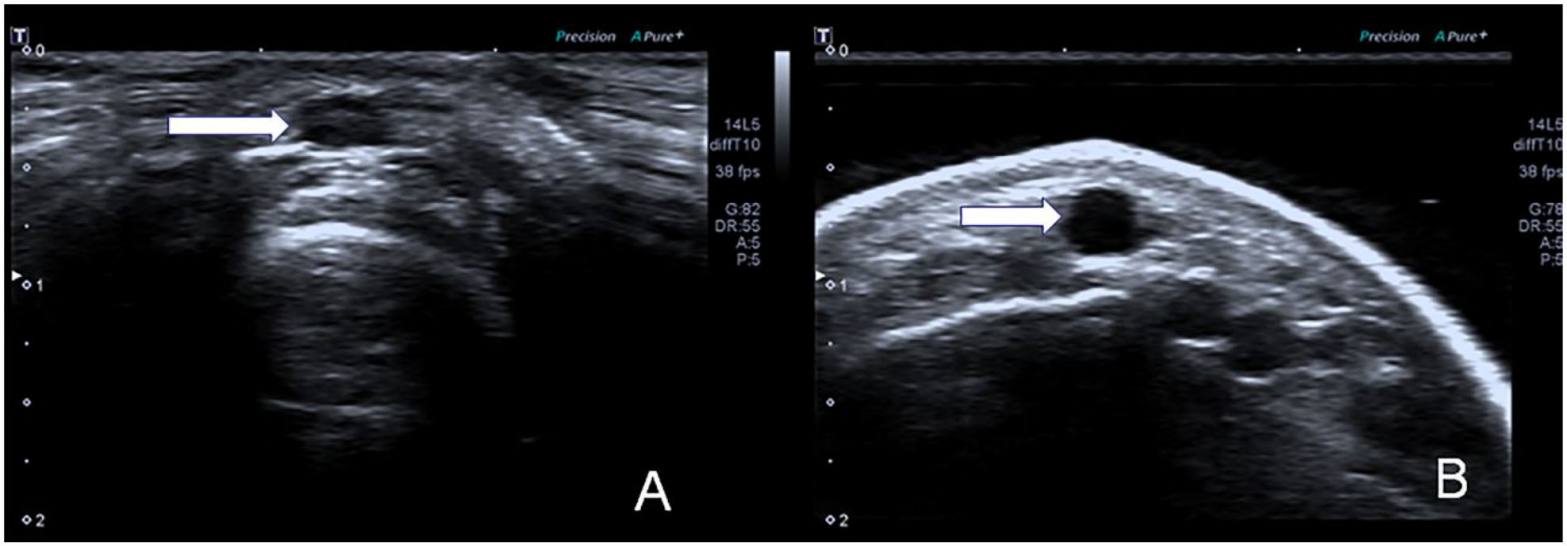
Ultrasound images of the cephalic vein (arrow) displayed *via* two approaches. (A) Contact ultrasonography. (B) Immersion ultrasonography.

Immersion ultrasonography of the cephalic vein provides two-dimensional images with a wider display that more easily includes both the cephalic vein and radial artery (Figure 3).

## Discussion

The efficacy of ultrasound for measuring the cephalic vein diameter before AVF creation has been proven, but the reproducibility of the measurements remains controversial [2]. Different operators will obtain different measurement results, with a strong dependence on the experience of the operators.

Because of the superficial position of the cephalic vein in the wrist, the vein can easily be deformed by pressure from the probe, resulting in measurement error. Previous literature has shown [9] that the cross-sectional area of the cephalic vein is related to the pressure and that different pressures produce different cephalic vein shapes. Whether the examination is performed by the same operator or by different operators, at the time of every cephalic vein measurement, use of the same pressure cannot be ensured, and the pressure is influenced by external factors. The literature has shown that the lack of a quantitative index of probe pressure applied by the operator leads to variation in the results [10] and that probe pressure affects the blood flow velocity in the tissue [11]. Even if a thick layer of coupling agent is applied to reduce compression, indirect pressure on the cephalic vein is necessary to obtain a clear ultrasound image. During the operation, the probe needs to be suspended in the coupling agent, which causes fatigue in the operator’s arm and unstable imaging. In evaluating the cephalic vein, the probe must be moved to provide a comprehensive view of the cephalic vein; however, due to the uneven shape of the wrist, the probe is difficult to move, which increases the difficulty of the process. In immersion ultrasonography, the ultrasound probe does not directly contact the examination site; instead, liquid is used as the ultrasound medium, which can avoid the influence of probe pressure on the cephalic vein. Moreover, this approach avoids the influence of human factors, such as the strength and habits of different operators. In the context of ocular diseases, immersion ultrasonography measurements have been reported to exhibit good accuracy and reproducibility, and immersion ultrasonography has been reported to yield more accurate axial length measurements than contact ultrasonography [12], with very high accuracy [13,14]. Immersion ultrasonography reduces the difficulty of the examination and does not require the operator to have extensive experience to ensure a lack of pressure on the blood vessels. Furthermore, in immersion ultrasonography, the probe can easily be moved to view the whole cephalic vein, which improves the repeatability of cephalic vein measurement by inexperienced operators.

It is important to clearly show the wall structure of the cephalic vein, which affects the exact location of the ultrasound scale and ultimately affects the accuracy of the measurement. It has been reported that it is difficult to locate the vessel wall in two-dimensional ultrasound images and that obtaining clear images may improve the repeatability of cephalic vein measurements [15]. Because the cephalic vein position is superficial and in the near field of the ultrasound beam, the quality of the tissue image is decreased, the vascular wall is not clearly displayed, and it is difficult for the operator to determine the scale position. Using immersion ultrasonography, the water medium increases the near-field distance so that the superficial cephalic vein can be localized in the focus area of the acoustic beam and its structure can be clearly displayed. It has been reported that increasing the near-field distance can improve the quality of grayscale sonograms of tissues within 1 cm of the body surface [14]. Compared with contact ultrasound, this approach allows the structure of the cephalic vein wall to be viewed more clearly, the measurement scale to be placed more accurately, and the consistency of repeated measurements to be increased. In addition, the authors found that the use of water as the ultrasound medium allowed the tissue to be shown over a wider area, which was helpful for viewing the relative positions of the cephalic vein and radial artery. Because the wrist skin is curved, the surface area of contact between the ultrasound probe and the skin is limited, and the skin cannot be completely fitted to the probe, resulting in a limited range of ultrasound images that can be displayed. Immersion ultrasonography uses water as the ultrasound medium, which completely fills the gap between the skin and the probe; thus, the entire ultrasound probe can be used to image the target, with a wider inspection range.

We recommend that inexperienced operators first understand the anatomical location of the cephalic vein and the ultrasound images before performing immersion ultrasound, so that the operator can complete the operation independently. Only the front end of the ultrasonic probe is immersed in the water, and the part of the probe the operator holds is above the water surface. This not only facilitates movement of the probe, but also keeps the operating environment relatively clean. When scanning the cephalic vein in water, try to keep the ultrasonic probe at a distance of approximately 1 cm from the skin of the wrist to avoid compressing the cephalic vein and to also to show more soft tissues around the cephalic vein.

It is worth noting that, in immersion ultrasound of the cephalic vein, it is sometimes unnecessary for the operator to move the probe because we can instruct the patient to reposition the arm to obtain an image of the cephalic vein at the desired position. The patient can reposition the arm so the operator does not need to move the probe, which greatly reduces the operator’s workload, ameliorates the difficulty of the operation, and ensures the accuracy of the measurement.

Immersion ultrasonography has some limitations. It is not suitable for patients with contraindications to fluid contact, e.g., broken forearm skin or severe skin disease. Although increasing the near-field distance can improve the image quality of the tissue within 1 cm of the body surface, the method has certain limitations with respect to the displayed image quality and measurement repeatability in obese patients.

In summary, the authors suggest the use of immersion ultrasonography for inexperienced operators to measure the cephalic vein diameter, as it can reduce the dependence of measurement on operational experience and achieve very good repeatability. The main reasons are as follows: (1) In immersion ultrasonography, the probe does not contact the skin, avoiding probe pressure on the blood vessel. (2) The use of water as the medium increases the cephalic vein near-field distance, improves the image quality, and increases the accuracy of the measurement scale position. Therefore, for measurement of the cephalic vein diameter by inexperienced operators, immersion ultrasonography can reduce experience dependence and yield very good repeatability and is thus worthy of promotion.
